# Targeting Endothelin in Alzheimer's Disease: A Promising Therapeutic Approach

**DOI:** 10.1155/2021/7396580

**Published:** 2021-09-06

**Authors:** Shiwali Sharma, Tapan Behl, Anoop Kumar, Aayush Sehgal, Sukhbir Singh, Neelam Sharma, Saurabh Bhatia, Ahmed Al-Harrasi, Simona Bungau

**Affiliations:** ^1^Chitkara College of Pharmacy, Chitkara University, Punjab, India; ^2^Delhi Pharmaceutical Sciences and Research University, Delhi, India; ^3^Natural & Medical Sciences Research Centre, University of Nizwa, Nizwa, Oman; ^4^Amity Institute of Pharmacy, Amity University, Haryana, India; ^5^Department of Pharmacy, Faculty of Medicine and Pharmacy, University of Oradea, Oradea, Romania

## Abstract

Endothelin is a chemical mediator that helps in maintaining balance within the blood-brain barrier by regulating the levels of toxicants and molecules which pass through the brain, suggesting that a rise in its production determines Alzheimer's disease. The inequity in the amyloid *β* occurs due to a problem in its clearance from the brain initiating the production of reactive oxygen species and superoxide that activates a cascade wherein the release of inflammatory mediators and various enzymes like endothelin-converting enzymes take place. Furthermore, the cascade increases the levels of endothelin in the brain from endothelial cells. Endothelin levels are upregulated, which can be regulated by modulating the action of endothelin-converting enzymes and endothelin receptors. Hence, endothelin paves a pathway in the treatment of Alzheimer's disease. In this article, we have covered various mechanisms and preclinical studies that support and direct endothelin involvement in the progression of Alzheimer's disease by using various search tools such as PubMed, Science Direct, and Medline. Conclusive outcome data were extracted that all together defy contrivance pathways, potential drugs, endothelin receptors, and endothelin enzymes in our article giving profound importance to target endothelin for prevention and treatment of Alzheimer's disease.

## 1. Introduction

Alzheimer's disease can be identified by manifestations like atrophy and fragmentation of blood vessels, decreased density of microvasculature, irregularities in capillaries, change in the diameter of blood vessels, and increased thickness due to elevated collagen of the basement membrane. These progressive changes lead to the formation of neurofibrillary tangles or senile plaques and inadequate blood flow leading to cognition and memory loss [1–3]. The striatum is the region of the brain wherein the early detection of amyloid *β* deposition can be seen [4]. It is ubiquitously estimated in 24.3 million people in 2005 all over the world and is predicted to rise to 81.1 million in 2040 if the permanent therapy for preventing or curing the disease is not found [5]. It is the fifth leading cause of death worldwide affecting persons with age groups more than 65 years of age. Moreover, recent research has concluded that most patients have vascular deformities [6, 7]. Alzheimer's occurs in a late stage of life, and its ubiquity doubles every five years after 65 years of age. Hence, considered a complex disorder, Alzheimer's is classified majorly into two types: sporadic form (affect individuals more than 60 years of age) and familial form (occurs at an early age mostly between 30 and 50 years with rare genetic mutations) [8]. Multiligand receptors like a receptor for the advanced glycation end-product (RAGE) belong to the immunoglobulin superfamily of cell surface molecules and are found on glial and endothelial cells involving the development of Alzheimer's disease. The development is due to amyloid *β* cytopempsis across the blood-brain barrier through a receptor for advance glycation, where the end-product amyloid *β* is taken up by neurons leading to cellular stress and expression of the large amount of endothelin 1 in the brain, thereby causing reduced cerebral blood flow [9]. Vascular dysfunction is the initial step in the progression of Alzheimer's leading to oxidative stress, damage of nucleic acids, proteins, and lipids and peroxynitrites such as superoxide and nitric oxide directly interfering in nitric oxide production through tetrahydrobiopterin cofactor; the absence of this enzyme results in the formation of reactive oxygen species [2]. The haemostatic cerebral blood flow is regulated and balanced by arterial blood pressure, and the metabolism is maintained by an equilibrium between demand and supply in energy towards CNS (central nervous system) and neurogenic autoregulation, which modulates vascular tone in the peripheral and central nervous systems. Therefore, all these mechanisms work together with the neurovascular units which involves glial cells, perivascular nerves, and vascular cells [10]. The cerebral blood flow is an autoregulated mechanism where cerebral perfusion pressure is regulated by vasomotor effectors and increases the blood flow that further releases vasoactive agents such as endothelin 1 (ET1), vascular endothelial growth factor (VEGF), and angiotensin-converting enzymes. This produces cerebral ischemia that generates a pathological condition of vascular dementia causing Alzheimer's disease [11, 12]. In vitro studies have proved transgenic mice with increased levels of amyloid *β* having impaired cerebral blood flow [13, 14]. There is dysregulation of the endothelin system and reduced cerebral blood flow up to 40 percent that is due to cerebral amyloid angiopathy and nonamyloid small vessel disease, causing a decrease in cerebrospinal fluid in the brain having Alzheimer's disease [15–17]. Studies have proved that amyloid *β* interacts with endothelial cells in blood vessels that generate superoxide radicals, which can find endothelium-derived relaxing factors and generate potent oxidizing agents, thereby increasing the lipid peroxidation and forming deformative functions and structures of the endothelium [18, 19]. Reactive oxygen species are among the (ROS) prominent reasons for the dysfunction of cerebral vasculatures in brain-related disorders. Studies were done to illustrate the impact of reactive oxygen species (ROS) where samples of endothelial cells were taken from the brain of Alzheimer's patients and healthy people and found that increased oxidative stress in the high age group of Alzheimer's patients correlates with decreased arterial vasodilation. It has been concluded by various studies that there is a high impact of oxidative stress on cerebral vasculature which is increased by the effect of nuclear factor kappa light chain enhancer (NF-*κ*B) damaging the vascular endothelium by releasing cytokines [20]. Endothelial dysfunction acts as a molecular target for neurodegenerative diseases like Alzheimer's as it acts through a reversible process [21, 22].

## 2. Occurrence, Synthesis, and Functions of Endothelin

Endothelin (ET) is also known as an endothelium contracting factor that comprises of distinctive peptides of 21 amino acids, and its basic structure was studied by isolating its isoforms known as porcine or human endothelin (ET1), endothelin 2 (ET2), rat endothelin 3 (ET3), and vasoactive endothelin constrict (VIC) or polypeptide or mouse, murine (VIF). These peptides comprise of disulphide bond that is present in the loop like structure with C terminal hydrophobic tail having 21 positions occupied with tryptophan [23–25]. Biosynthesis occurs from preproendothelin to proendothelin also known as big endothelin which breaks to form mature endothelin [26]. Endothelin 1 is wholly produced by vascular endothelial cells whereas endothelin 2 and endothelin 3 are present in nonvascular cells in various organs like the kidney, brain, and adrenal gland [27]. ET3 is particularly present in neural tissues having a role in cell generation and growth; however, endothelin 2 is mainly found in the intestine and kidney. Endothelin (ET) is formed in the brain by neural, nonneural, or vascular elements or directly produced in cerebrospinal fluid [28]. ET1 plays a pivotal role in generating inflammatory processes due to an increase in brain amyloid *β* proteins which has been proven in preclinical studies done on an animal model of rats having Alzheimer disease where administration of amyloid *β* spikes levels of ET transduction mechanism, cognitive impairment, and oxidative stress [29]. Loss of neurons in the brain of Alzheimer specifically in the cortex part decreases endothelin binding sites [13]. ET1 increases various cell adhesion molecules 1 (CAM-1), vascular cell adhesion protein 1 (VCAM-1), and e-selectin in human brain vasculature consisting of endothelial cells projecting towards infiltration, margination, and adherence in endothelial cells and injured tissue where platelets get accumulated hence inflammatory cascade formed causing vascular dysfunction [29, 30]. Studies have shown that intrahippocampal injection of endothelin and aggravated amyloid *β* (1-40) predominantly increases neuronal death in the dentate gyrus and increases proinflammatory cytokines in the hippocampus of the brain causing memory loss and cognitive impairment [31]. Endothelin 1 (ED1) functions as a vasoconstrictor, and it is mainly found in brain neurons, smooth muscle cells, vascular endothelin, and macrophages [32]. Endothelin 1 precursor fragments improve the diagnosis of Alzheimer's disease [33]. C-terminal proendothelin-1 (CT-pro-ET1) is a functionally inactive surrogate of endothelin 1 precursor fragment that can lead to Alzheimer's disease [34]. ET1 production takes place from the endothelin 1 precursor gene (EDN1) by transcription and translational process, and it enters into the endoplasmic reticulum before ED1 secretion from the cell furin like proteases that interact with preproET1 resulting in the formation of big ET1 which is inactive until it interacts with endothelin-converting enzyme (ECEs) to form active ET1 [5]. ET1 has many functions like cell proliferation, inflammation, and cell coagulation. It is released in response to the decreased potential of hydrogen (PH), angiotensin II, thrombin, and plasminogen activator inhibitor 1 (PAI-I), and due to the autocrine function of EA2 [35]. Endothelin 1 decreases the vessel lumen increases vascular resistance and results in vascular insufficiency [36].

## 3. Significance of Endothelin in Alzheimer Disease

**Figure 1:**
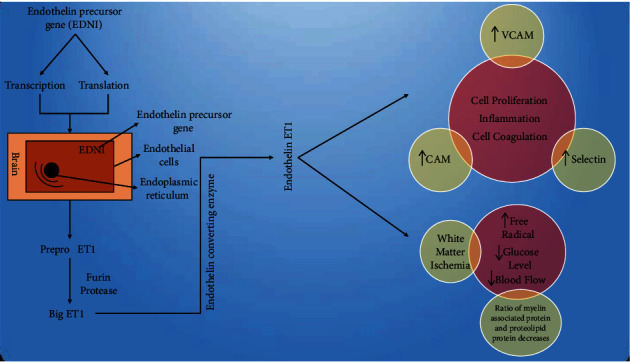
Endothelin precursor gene present in the human brain by transcription and translation processes leads to the formation of endothelin, and its rise causes an increase in vascular cell adhesion protein (VCAM), cell adhesion protein (CAM), selectin, white matter ischemia, decrease in the ratio of myeline-associated protein and proteolipid protein increasing various factors like free radical formation, inflammation, cell coagulation, cell proliferation, decrease glucose level, and blood flow directing towards the pathogenesis of Alzheimer disease.

ET1 is an proinflammatory cytokine which is abundant in fibroblast, macrophages, and leukocytes and hence defines its role in inflammatory processes. ET1 releases cytokines from mast cells. Platelets are aggregated due to the action of ET1 which accumulates several inflammatory mediators and leukocyte adhesion molecules, causing vascular dysfunction [37]. Studies have proved that intrahippocampal injection of endothelin 1 (ET1) causes neurodegeneration and loss of cells in the pyramidal cell layer [4]. Endothelin 1 (ET1) results in white matter ischemic damage that results in decreased blood flow. Studies have concluded that the brain of Alzheimer's patients has an increased amount of endothelin 1 (ET1) in the temporal cortex of the brain [12]. There are prominent findings through various studies that arteriolosclerosis small vessel disease clears interstitial solutes like amyloid *β*. Endothelin 1 (ED1) gene is found to be double in the fold of concentration in the early diagnosis of Alzheimer's disease [15]. Endothelin 1 (ET1) has a role in regulating blood flow to the heart and other local organs. Neurological damage is produced by reduced blood flow due to contraction in cerebral arteries and arterioles causing apoptosis and microinfarcts and neuronal death-causing early prognosis in the development of Alzheimer's disease [13]. Transforming growth factor *β*1 transgenic mouse model of Alzheimer's disease has found that vascular deformity occurs due to endothelin 1 (ET1) [38]. Another study has explained the role of endothelin 1 (ET1) in Alzheimer's disease under in vitro experiment where endothelin 1 (ET1) (1 Nm) was given as a pretreatment drug in a neuronal culture that causes neuronal damage which was found like damage produced by thrombin [39]. The endothelium degeneration occurs with the progress of age due to thromboxane, prostaglandins, and prostanoids responsible for inducing inflammation [20]. Studies on immunohistochemical analysis have proven that Alzheimer disease patient has high levels of ED1 in the cerebral cortex of the brain. Preclinical studies in animals were done where ED1 was applied to cerebral arteries or given by intracerebral injection produced vasoconstriction, and hence, focal necrosis was observed [39]. Endothelin functions as a vasoconstrictive agent and helps in stimulating cell division in astrocytes and tumour cells when studied in in vitro models signifying its role in modulating the neuronal activity of the central nervous system [40]. Removal of toxic proteinaceous molecules like amyloid *β* by cytopempsis through receptors across the blood-brain barrier is difficult and a study of a mathematical model concluded that it takes 13 minutes under physiological conditions for amyloid *β* to get removed from the brain through the transport of the blood-brain barrier into the blood with the help of low-density lipoprotein receptor-related protein 1 (LRP1) when production of amyloid *β* is from amyloid *β* precursor protein and circulating amyloid *β* transport is restricted from the brain this signifies the importance of the transvascular system which does not allow transport of toxic, accumulated amyloid *β* into the brain [41]. Preclinical studies show that endothelin 1 (EDN1) produces amyloid *β* by stimulation of amyloid *β* receptors for RAGE leading to decreased cerebrospinal fluid in the Tg2576 mouse model of animals. Elevated EDN1 is found in the cerebral cortex of Alzheimer's [16] (Figure 1).

## 4. Role of Endothelin Receptors in Alzheimer Disease

### 4.1. Endothelin Receptors (ETA and ETB)

There are two major types of subreceptors of endothelin: endothelin A receptor (ETA) and endothelin B receptor (ETB) that belong to the G-protein-coupled receptors having seven transmembrane domains that contains 22 to 26 amino acids having a hydrophobic bond. Many actions are produced by the activation of endothelin receptors; one such pathway explains that ETA activates phospholipase C that results in inositol triphosphate and diacylglycerol from phosphatidylinositol after that inositol 1,4,5 triphosphate (IP3) diffuses into specific receptors that are present on the endoplasmic reticulum releasing calcium into the cytosol, elevating its level and causing cellular contraction and vasoconstriction [42, 43]. Endothelin A receptors are present in pericyte cells where they get activated with a rise in endothelin 1, and this mechanism has been proven by various studies performed on the postmortem brain of an Alzheimer patient [44]. It was found that deletion mutation in endothelin-converting enzyme 1 (ECE1) and endothelin-converting enzyme 2 (ECE2) increases amyloid *β* proteins that are A*β* (1-40) and A*β* (1-42) proved by the animal model (rat) of Alzheimer disease in addition to this increased level of ECE2 in the brain altogether indicates a clear pathway towards Alzheimer disease. Endothelin has neuropeptide substrates such as substance P, neurotensin, and bradykinin [45]. Endothelin 1 (ET1) and endothelin 2 (ET2) get attached to endothelin receptors which are G-protein-coupled receptors in their origin [46, 47]. Endothelin 1 (ET1) dysfunction generates superoxide by activation of ETA and ETB receptors, the process is further initiated by the formation of peroxynitrite that downregulates the formation of nitric oxide, and hence, the inflammatory process and oxidative stress are initiated leading to vascular endothelial dysfunction. Preclinical studies have proven that when endothelin receptors are antagonized using drugs such as bosentan they decrease inflammatory mediators and vascular dysfunction in brain carotid arteries hence maintaining cerebrospinal blood flow and are efficient in Alzheimer's with vascular dementia [48]. Drugs that are used in the improving vasospasm and decreased blood flow target ETA receptors in the brain [49]. Endothelin functions in regulating the brain and systemic blood flow through ETB receptors widely distributed in the brain. Studies on the human brain with Alzheimer's disease were done that have proved ET1 to be present in the occipital and frontal lobes of the cerebral cortex that enters vascular smooth muscle cells and leads to reduced outflow of blood towards the brain due to vasoconstriction [50]. Increased amyloid *β* does not have any effect on endothelin receptor B (ETB); therefore, targeting endothelin receptor A does not show any effect on these receptors. More ET binding sites are present on the ETB receptor [13]. IRL-1620 selective agonist stimulates endothelin receptor B. Previous research on endothelin B receptors has proved that when the rats' central nervous system has less number of these receptors they produce a large amount of endothelin 1 within the cerebrovascular system causing vasoconstriction, apoptosis, and lowering down several neural progenitor cells [51]. Endothelin receptor agonist IRL-1620 can help in treating various CNS neurological disorders. Preclinical studies of Alzheimer's disease using animal models of rats were performed to know the potential of IRL-1620, and it was concluded that it improved cognitive activity, and it further decreased oxidative stress and increased VEGF and NGF in amyloid *β*-treated rats by direct stimulation of endothelin receptor B when given intravenously. Another study on the APP/PSI transgenic animal model of the mouse of Alzheimer's disease when treated with IRL-1620 may improve memory functions and activity. So, it was concluded that IRL-1620 may cause angiogenic remodeling therefore effective in Alzheimer's disease [52]. Endothelin (ET) antagonists block stimulation of endothelin receptors caused by amyloid *β* in the hippocampus, brain stem, and cerebral cortex but do not affect other parts of the brain. Tezosentan is an ETA/ETB receptor antagonist. BQ123 and BMS 182874 reduce oxidative stress, the main cause of Alzheimer's disease, and it is an ETA receptor antagonist [13]. Different hypothalamic nuclei mediate endothelin receptors to function in neuroendocrine modulation [53]. Vascular endothelial cells produce vasoactive protein endothelin 1 that leads to inflammation in the vascular system of the brain [39]. In vitro studies on TGF aged mice have used ABT-627 drugs that act as an antagonist of endothelin A receptor (ETA), and it was found that this ABT-627 antagonist hinders the vasospasm activity of endothelin 1 (ET1) and nitric oxide synthesis. This mechanism of ABT-627 is due to its action on activated astrocytes [54]. Blocking endothelin receptors is a great treatment for vascular and nonvascular diseases. An increase in endothelin leads to endothelin cell stress reaction leading to contraction, denudation, and exuviation of endothelial cells in Alzheimer's disease. Endothelial NOS precursors, vitamin E, and water-soluble vitamin C are considered a treatment or preventive measure for Alzheimer's disease [11, 55]. Constant activation of the endothelin receptor by endothelin 1 generates calcium dyshomeostasis that leads to neuronal damage which mimics Alzheimer's disease [56].

## 5. Role of Endothelin-Regulating Enzyme in Alzheimer's Disease

Endothelin-converting enzymes (ECEs) are from the group of MI3 zinc metalloproteases that get attached to endopeptidases which break the hydrophobic bond present at the amino acid. ECEs function by hydrolysis of inactive biological intermediates that are big endothelin ET1, ET2, and ET3 at the Trp21-Val/I1e22 bond. The interaction between endothelin receptors and endothelin takes place for performing their respective functions of endothelin enzymes [57]. Endothelin-converting enzymes (ECEs) have a cytosolic domain with an N terminal and catalytic domain that is present in the extracellular space. Phosphoramidon is an antagonist of endothelin-converting enzyme (ECE) that leads to the deposition of amyloid *β* in the cell line that expresses ECE that shows degradation action of endothelin-converting enzyme on amyloid *β*. Cell line studies have shown that overexpression of ECE decreases amyloid *β* up to 90 percent, and in vivo studies show that transgenic mice (ECE +/−) have a 25 percent decrease in endothelin-converting enzyme activity ultimately showing an elevated rise of amyloid *β* 40 and amyloid *β* 42 in the brain [58–60]. Findings from studies suggest that endothelin 1 (ET1) is increased in Alzheimer's disease because of increased activity of enzymes like endothelin-converting enzymes they convert big endothelin into ED1, hence decreasing blood flow towards the brain ultimately leading to the pathogenesis of Alzheimer's [39]. ECE1 and ECE2 can break amyloid *β*. The dissimilar alleles of ECE1 present in knockout mouse brain has an increased level of amyloid *β* 40 and amyloid *β* 42; further investigation through preclinical studies shows that endothelin-converting enzyme (ECE1) and endothelin-converting enzyme 2 (ECE2) concentrations were increased in small blood vessels in the brain of Alzheimer disease patient; more illustrations were done through further studies, and it was investigated that amyloid *β* 42 and oxidative stress are key players for protruding the release of endothelin 1 from brain endothelial cells [5, 61]. Amyloid *β* and endothelin 1 (ET1) are products that are produced by regulating the human endothelin-converting enzyme (ECE) and ECE1 human gene [62]. The role of the endothelin-converting enzyme is to convert endothelin to its active form and hence regulate blood flow [60]. Protein kinase C is the enzyme that is known to regulate the activity of endothelin-converting enzymes (ECEs) in the brain and helps in regulating the clearance of plaque in the brain proved by in vitro studies on mice; this is known to occur due to the overexpression of protein kinase [63]. Amyloid *β* is considered a physiological substrate for the endothelin-converting enzyme (ECE). In vitro studies have proved that animals deficient in endothelin-converting enzyme have cognitive and memory impairment [64]. An increase in amyloid *β* occurs in Alzheimer disease that tends to protect the brain from reduced blood flow toward the brain. The overactive endothelin-converting enzyme 1 and 2 from neuroblastoma and endothelial cells of the human brain elevates the level of ED1. EDN1 is increased in the brain cerebral cortex with Alzheimer's disease; the precuneus is the starting point from where the decline of blood flow starts. Amyloid *β* 40 and amyloid *β* 42 upregulate EDN1 and hence increase in ECE1 and ECE2, respectively, in endothelial cells [65]. Zinc metalloproteases play a role in amyloid *β* turnover in the central nervous system that further modulates protease activity of endothelin-converting enzyme directly linking an endothelin role in Alzheimer disease [66].

### 5.1. Endothelin-Converting Enzyme 1 (ECE1)

ECE1 is present in endothelial cells of all organs including neuroendocrine, neurons, and glial cells. Studies show that the ECE1 enzyme can act as a preventive measure against Alzheimer disease which is proved by studies done on Chinese hamster ovary cells where it was found that cells that have an overexpression of the ECE1 enzyme and lack endogenous ECE activity have the potential to reduce up to 90 percent amyloid *β* concentration; this effect can be antagonized by using the ECE1 inhibitor phosphoramidon. The endothelin-converting enzyme 1 (ECE1) activity affects the concentration of amyloid *β* directly by decreasing its concentration. Studies show that a minimum of three sites of amyloid *β* can be cleaved by ECE1 that forms fragments of amyloid *β* 1-16, 1-19, 1-17, and 20-40 [37, 67]. ECE1 is present in the brain and produces endothelin. ECE1 is involved in endosomal neuropeptide metabolism and helps in regulating the recycling of peptide receptors [68]. ECE1 is increased in endothelial cells by amyloid *β* 40 [16, 69]. Amyloid *β* is metabolized by ECE1. Studies show that cultured hamster ovary cells have an ECE1 activity but do not show ECE function, and hence, up to 90 percent of amyloid *β* is reduced; this occurs through cleavage by ECE1 in three sites of amyloid *β* to form its fragments: amyloid *β* 1-17, amyloid *β* 1-19, and amyloid *β* 20-40 [70]. Protein kinase enzymes maintain the level of ECE in the brain [63]. Neprilysin and endothelin enzyme 1 (ECE1) knockout animal model of mice concluded that there was a rise in amyloid *β* [71].

### 5.2. Endothelin-Converting Enzyme 2 (ECE2)

**Figure 2:**
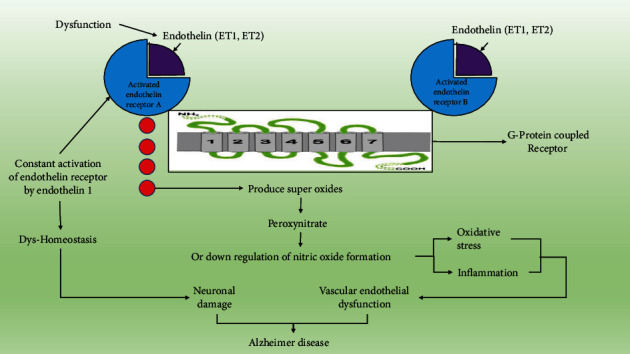
Mechanisms of dysfunction in endothelin where constant activation of endothelin receptor A and endothelin receptor B leads to neuronal damage and vascular dysfunction indicating endothelin to be a potential target for Alzheimer's disease.

Endothelin-converting enzyme 2 is a substrate-specific enzyme and processes at nonclassical sites that are in the intracellular surface which includes endosomes. It shows optimum activity at pH 5.5 and is known to show activity in intracellular compartments which are restricted in the neuroendocrine system. Studies have found that enzyme ECE2 has a significant role in the degradation of amyloid *β* as it has a significant role in Alzheimer's disease [60, 72]. ECE2 is present in somatostatin expressing (SOM+) interneurons that have a role in cognitive function. Amyloid *β* accumulation is toxic to SOM+ interneurons induced by ECE2 mutation; this leads to loss of SOM+ interneurons and deficit in cognitive function [57]. Lack of ECE2 showed a gene dose effect on amyloid *β* accumulation [73]. ECE2 is found locally in pyramidal cells of the human brain, when there is an accumulation of amyloid *β* 42 and not amyloid *β* 40 [74]. Studies were done with the use of WES technology; it was identified that ECE2 R186C mutation segregation was done with Alzheimer disease phenotype with one branch of LOAD pedigree where ECE2R186C was found as an Alzheimer disease-associated gene variant. It was found that R186C mutation impaired endothelin-converting enzyme 2 (ECE2) ability to degrade amyloid *β*. The sequence kernel association (SKAT-O) test showed that nonsynonymous rare coding mutations in the endothelin-converting enzyme 2 (ECE2) gene was in abundance in Alzheimer's patients when compared to the control. The R186C mutation gene or F751S that was found in the patient with Alzheimer's disease is present in the MI3 active peptidase domain. Overexpression of ECE2 has enough amyloid *β* degradation property to decrease amyloid *β* 40 and amyloid *β* 42 levels. Further in vitro analysis demonstrated that R186C or F751S mutant removed the activity of ECE2 that lead to impaired degradation of amyloid *β*. In vivo, preclinical studies on mouse models showed that R186C mutation caused the loss of ECE2 function that results in finding that ECE2 R186C proteins may lead to amyloid *β* accumulation forming neuritic plaque and cognitive deficits [57]. EDN1 is stimulated by ECE2 and progressed by amyloid *β* 40 plays a vital role in vascular dysregulation in Alzheimer's disease [36]. ECE2 is largely found in the central nervous system and found to be active intracellularly on the surface of the cell [75]. Studies show that ECE2 knockout mice have amyloid *β* 1-40 and amyloid *β* 1-42 regulating intracellular pool of amyloid *β* peptide [68]. Amyloid *β* 42 increases ECE2 in endothelial cells [18]. ECE2 mRNA and protein levels are elevated in the temporal neocortex in Alzheimer disease; further, this has been proved by in vitro studies on SH-SY5Y where treatment with oligomer amyloid *β* 1-42 for 24 hours increases ECE2 mRNA, and the ECE2 gene response is upregulated in response to amyloid *β* [76]. Studies have found that ECE2 knockout mice have a critical response towards SOM+ interneurons that are responsible for cognitive function and are approximately 5 to 8 percent of the total population, hence showing similarity with pathological conditions of Alzheimer's disease where complete loss of SOM interneurons take place [77]. There is a reduction of endothelium-converting enzyme 2 (ECE2) from parietal lobe tissue in the Alzheimer's brain [78]. Elevated activity of endothelial enzyme 2 is considered a compensatory mechanism to decrease the level of brain amyloid *β* proteins. ECE2 suppresses P-glycoprotein-1 in endothelial cells of the blood-brain barrier, therefore affecting the amyloid *β* transport [79] (Figure 2).

## 6. Endothelin Mechanism Contributing to Alzheimer's Disease

### 6.1. Endothelin Decreases Blood Flow in the Brain

Amyloid *β* causes an increase in the production of neurons that produces endothelin 1 (ET1) by upregulating endothelin-converting enzyme 2 (ECE2) changing ET1 from an inactive precursor to active form; this upregulation of endothelin 1 and endothelin-converting enzyme axis due to amyloid *β* 1-40 causes chronic reduction of the cerebral blood flow in Alzheimer disease. Studies have proved that the treatment of L-N nitro arginine in the human (L-NAME) brain increases amyloid *β* precursor and *β* site amyloid precursor cleavage enzyme that is beta-secretase 1 (BACE-1) which uplifts the total level of amyloid *β*. In concurrent to this, in vitro studies done on mice with deficient endothelin nitric oxide synthase (eNOS) have also suggested that their brain and microvessels contain a large amount of amyloid-beta precursor protein and beta-secretase enzyme activity with total amyloid *β* protein when compared to wild-type control mice [80]. Oligomeric amyloid *β* is responsible for activating inflammatory mediator interleukin-1*β* is generated in the microglia and astrocytes which release endothelin 1 and decrease the cerebrospinal blood flow that makes neurodegeneration in the brain causing Alzheimer's disease [44]. Efflux of amyloid *β* depends on low-density lipoprotein receptors of the endothelium. Amyloid *β* has a toxic effect on endothelial cells by a direct and indirect mechanism; in vitro studies show the toxic effect of amyloid *β* (1-42) on endothelial cells where it increases apolipoprotein E4 and decreases apolipoprotein E2 [75]. Studies have proved that amyloid *β* potentiates the constriction of endothelin 1 when it was applied to cerebral arteries of the human brain after rapid autopsy; this occurs due to an enhanced proinflammatory mechanism [81]. Studies prove that overexpression of ET1 in endothelial cells leads to cognitive impairment after reperfusion and short-term ischemia revealing its role in amyloid *β* and dementia; it was also found in another study that levels of ET1 mRNA are induced in astrocytes showing its role in Alzheimer [18]. Preclinical studies suggest that there is hypotonia of blood vessels that occurs in the striatal circulation of rats with immediate fall of the cerebrospinal blood flow when rats are given ET1 injection in the lesion part of ET1 or amyloid *β*+ET1 rats [82]. As indicated by previous in vitro studies, amyloid *β* has directly interfered with the division and migration of endothelial cells; the mechanism behind this action is the generation of reactive oxygen species in vascular cells which produce stimulation of enzymes like caspase, mitochondrial deformities, DNA dysfunction, and oxidative stress that ultimately leads to endothelial autophagy and apoptosis [83]. Preclinical studies suggest that oxidative stress is caused by an imbalance of amyloid *β* which causes vasospasm in the brain capillaries by accumulation of reactive oxygen species due to enzyme nicotinamide adenine dinucleotide phosphate oxidase. After this process, reactive oxygen species release ET1 which attaches to endothelin A receptors inducing vasospasm in pericytes and capillaries and hence generating a reduced cerebral blood flow leading to neurodegeneration and dementia and ultimately Alzheimer disease [84]. Endothelin 1 is responsible for controlling various astroglia functions like controlling various ion channel activity, secretion and uptake of glutamate, utilization of glucose, the permeability of gap junctions, and signaling of calcium. Astrocytes can produce endothelin 1 and contribute to its homeostasis in the central nervous system. Astrocytes also express endothelin receptor 1 and endothelin receptor 2 [85]. When physical and functional changes occur in astrocytes concerning pathogenesis, then, it is termed as a reactive astrocyte whose function is the overexpression of intermediate filaments like vimentin, glial fibrillary acidic protein, and reexpression of nestin that is mostly expressed in immature astrocytes. Larger bodies are the prominent feature of reactive astrocytes; these intermediate filaments have a role in neurological functions [86]. A high amount of amyloid *β* occurs in Alzheimer's disease which affects endothelial cells predominantly, and as endothelial cells age, its susceptibility towards the toxic effect of amyloid *β* also increases [87]. Studies have found that amyloid *β* 40 and amyloid *β* 42 increases vascular contraction by increasing endothelin 1 (ET1) expression in rat aortic rings that concluded that amyloid *β* decreases blood flow in the brain increasing amyloid *β* accumulation that leads to vessel degeneration and hence neurodegeneration [57]. In vivo and in vitro experiments have proved the role of the amyloid *β* protein in the production of vascular dysfunction by generating the production of endothelin through a direct mechanism without the involvement of mechanisms like cellular changes and apoptosis; it is known that this could occur due to the free radical effect of the amyloid *β* protein; this was further proved by experimental studies where A*β* 1-40 and A*β* 25-30 put on direct contact up to 24-48 hours with isolated vessels from the brain mainly cerebral arteries resulted in the contraction of brain smooth muscles and lead to vascular dysfunction [5]. Astrocytes play an implicate role in normal physiology by clearing and degrading amyloid *β* with the help of proteolytic enzymes such as endothelin-converting enzymes, neprilysin (NEP), insulin-degrading enzyme (IDE), and matrix metalloproteases that themselves are generated inside astrocytes [86]. Activated astrocytes and microglial act as chemokines and cytokines in the brain of Alzheimer patients. In vitro experiments show that when cultured endothelial cells were exposed to oxidative stress, a neurotoxic protease was released known as thrombin; this neurotoxic protease was also observed in senile plaque and neurofibrillary tangles (NFT), and in the cerebral vasculature in the progression of this, a response is produced for low cerebral blood flow in which hypoxia-inducible factor 1 alpha, monocyte chemoattract protein (MCP-1), interleukin 6 (IL-6), matrix metalloproteases-2, and reactive oxygen species (ROS) are upregulated [88]. In Alzheimer's, astrocytes become reactive and release endothelin 1 in the brain that leads to constriction in cerebral arteries, hence decreasing the flow of blood in the brain [89]. Astrocytes that are present in the reactive state in the pathogenesis of Alzheimer's disease release endothelin 1 (ET1). The brain vascular endothelial cell has low permeability towards various components of the blood but this is increased by activation of stimulated astrocytes releasing factors that produce various inflammatory mediators and endothelin 1 (ET1) from stimulated astrocytes [45]. Immunoreactivity of astrocytes towards endothelin 1 can be seen in the folia of the cerebellum and cerebral hemisphere with the white matter; after which, endothelin 1 oozes out of astrocytes and enter the microvasculature of the brain that decreases cerebrospinal fluid [53]. In vitro studies have proved that, in Alzheimer's brain, astrocytes are responsible for the production of endothelin 1 (ET1) that reaches the brain and leads to vasoconstriction in the vasculature of the central nervous system resulting in decreased cerebral blood flow. In response to damage to the central nervous system, a nonspecific reactive change occurs in glial cells that are induced by endothelin released by reactive astrocytes of the central nervous system. In vitro studies were done where endothelin 1 and endothelin 3 was directly put in contact with reactive astrocytes in response to which this endothelin increases the number of astrocytes by process of mitosis, and upregulation of phospholipids was also observed; hence, a conclusive result was found which demonstrates that endothelin increases the number of reactive astrocytes causing Alzheimer diseases [40]. Both cell adhesion-dependent and cell adhesion-independent mechanisms are involved in the activation of active astrocytes from endothelin in which the independent mechanism regulates active astrocytes from various extracellular factors that balance enzymes like kinase and protein kinase C affecting the expression of cyclin D1 in astrocytes, whereas in the cell adhesion-dependent mechanism, endothelin activates tyrosine kinase specifically activated focal adhesion kinase (FAK) [45]. Astrocytes are the target cell population of endothelin 1 in the brain. In vitro analysis of cultured astrocytes has shown the role of the extracellular signal-regulated kinase (ERK) and c-Jun N-terminal kinase (JNK pathway) are intricate in the pathway for cell proliferation and glial fibrillary acidic protein (GFAP). In vivo studies suggest that this pathway is also activated in reactive astrocytes as seen in enhance c-Jun N-terminal kinase phosphorylation which is prevented by Bosentan infusion [90].

### 6.2. Endothelin Generates Oxidative Stress

The release of endothelin 1 in the blood promotes free radical formation, and reduced blood flow and glucose levels can be seen by the decreased ratio between a myeline-associated protein with proteolipid protein 1 which increases the activity of ET1 in the brain [29]. Endothelin 1 is released by the effect of reactive oxygen species that lead to pericyte constriction by endothelin receptor A to cause vascular dysfunction in Alzheimer's disease [91]. The vascular structure of the brain vascular system consists of a neurovascular unit that is made of brain capillaries; in addition to this, the neurovascular system includes intracerebral arteries and small and large cerebral arteries. Mesenchyme homeobox gene 2 (MEOX2) is responsible for maintaining endothelium vascular functions; studies reveal that decreased levels of MEOX2 lead to abnormal response towards vascular endothelial growth factor that produces less developed vessels and disruption in the formation of blood-brain barrier including the decrease rate of cerebral blood flow. MEOX2 addition decreases the activity of LPR (lipoprotein density-related protein) that blocks the clearance of amyloid protein in the blood-brain barrier and enhances its accumulation in cerebral vessels leading to low cerebral blood flow and vascular dysfunction [92]. As the age of human progresses, changes that are both physical and chemical occur in vascular functions and gradually nitric oxide becomes less permeable to vascular structures of humans. This leads to free radical formation and less production of nitric oxide ultimately causing vascular stress, inflammation processes, and fluctuation in vasoconstriction. The endothelial nitric oxide synthase (eNOS) or ionic uncoupling agents generate ROS (reactive oxygen species) and stimulate vascular dysfunction which initiates from middle age. Parenchymal arterioles and large arteries produce vascular resistance in the brain that occurs in larger arteries having regular cerebral blood flow resulting in vascular endothelial dysfunction due to interfered neurovascular coupling commonly seen in women. Synthesis of nitric oxide occurs from arginine to citrulline into nitric oxide with the help of enzyme endothelin nitric oxide synthase involving tetrahydrobiopterin due to impaired endothelin function oxidation of this cofactor; tetrahydrobiopterin takes place making the change in a final by-product that form free radicals instead of nitric oxide [93, 94]. Nitric oxide synthase is an enzyme that constitutes of three members, where two are its isoform neuronal NOS (nNOS) and endothelial NOS (eNOS); both are calcium-dependent enzymes; another enzyme is inducible NOS (iNOS) and this is a calcium-independent enzyme. The NOS enzyme present in humans is linked to genes located on chromosome 7q 35-36 as 135 kDa in the endothelium. Expression of all these enzymes, endothelial nitric oxide synthase (eNOS), neuronal nitric oxide synthase (nNOS), and inducible nitric oxide synthase (iNOS), is increased due to the elevated process of tyrosine nitration in Alzheimer's disease. Studies show an abnormal expression of eNOS in the cerebral microvessels forming peroxynitrite which leads to a neurodegeneration disease known as Alzheimer's [95]. Endothelin is involved in the pathogenesis of Alzheimer's disease by producing PGE2 and nitric oxides from glial cells that take part in the inflammatory process leading to the accumulation of free radicals and hence degeneration of the brain activities and function in neurotransmission, neuromediators, and the neuroendocrine system. Experimental studies done preclinically shows that it stimulates the production of prostaglandins and thromboxane A2 from the rat or guinea pig isolated lungs whereas perfused peritoneum release nitric oxide due to the action of endothelin. In vitro studies on glial cell culture shows increased levels of prostaglandins E2 and nitric oxide because of all endothelin ET1, ET2, and ET3 in the brain; further findings suggest that these peptides are inflammatory mediators and result in brain damage where nitric oxide is a key player synthesized by endothelin which stimulates enzyme phospholipase C leading to arachidonic acid synthesis and releasing prostaglandin E2 or endothelin is also known to induce cyclooxygenase 2 in astrocytes, hence indicating direct neurotoxicity in the brain due to the role of endothelin in neurotoxicity with a high amount of endothelin in nerve cells [47, 96]. Endothelial nitric oxide synthase activation for a longer period leads to endothelial cell dysfunction, one of the dominant features of aging; this leads to the pathogenesis of Alzheimer disease with the formation of peroxynitrite causing accumulation of reactive oxygen species and nitrogen species around vascular endothelium. A low basal level of nitric oxide occurs that leads to an imbalance in endothelial cell conformation by thickening endothelial cells which is a compensatory mechanism for reduced nitric oxide as seen in Alzheimer's disease [97]. Vascular changes occur with age that affects the working of the brain; in the early stage of Alzheimer, the blood-brain barrier is interfered that occurs due to endothelial cells and cause an imbalance in the ratio of amyloid *β* entering the human brain producing reactive oxygen species (ROS) extracted by nicotinamide adenine dinucleotide opening transient receptor potential melastatin 2 channels which disrupt vascular function by increasing calcium load in brain endothelial cells that cause contraction [7].

### 6.3. Endothelin Produces Inflammatory Mediators and Angiogenesis

Endothelial cells and blood vessels which are unable to filtrate are the cause of inflammation particularly due to mechanical and biochemical damage in the blood-brain barrier [98]. Homeobox protein (MEOX2) in brain endothelial cells that lead to divergent blood vessels and abrupted response to vascular endothelial growth factor (VEGF) includes various angiogenic factors resulting in premature vessel regression after this amyloid *β* peptide with amyloid deposits get stick to an outer surface of basement membrane promoting inflammatory response that is local by activating perivascular microglia, astrocytes, and pericytes and then stimulating cerebral endothelium for production of proinflammatory mediators like vasoactive endothelin and chemokines that reduce cerebrospinal blood flow to brain increasing brain stress and leading to Alzheimer disease [99]. ET1 is a potent vasoconstrictor in smooth muscle cells that leads to focal ischemia and hence neuronal injury and directly acts on endothelial cells hence its possible role in Alzheimer's disease [39]. Hypoxia leads to ET1 production as it gets stimulated by hypoxia-inducible factor 1 *α*. ET1 is responsible for angiogenesis one of the cause of Alzheimer's disease. Higher levels of NAD(P)H oxidase are present in endothelial cells of the brain which makes them more responsive towards oxidative stress and hypoxia so the angiogenic factor is produced by endothelial cells of the brain towards hypoxia [100]. Endothelin helps in controlling the permeability of the brain helping endothelial cells and pericyte activity to have a coordinated activity with microvasculature present inside the brain [101]. Both hyperhomocysteinemia and amyloid *β* induces memory deficit, the most dominant feature of Alzheimer's disease by the degradation of the vascular endothelial pathway through upliftment of ET1 in the brain [102] (Table 1).

## 7. Conclusion

The current review concludes that endothelin can act as a potential target for the treatment of Alzheimer's disease by using its antagonist and agonist. Endothelin-converting enzymes and endothelin receptors are also targeted to find effective treatment and prevention of Alzheimer's disease. The role of endothelin receptor B is unexplored, and minimal research is done on its effect whereas many different studies are done on endothelin receptor A that mainly explains mechanisms like oxidative stress, inflammation, and others, so there is a need of exploring more mechanisms that are involved in the pathogenesis of Alzheimer's diseases. Targeting endothelin focuses on the neuroregeneration approach of treatment that may be a more beneficial way of controlling its pathogenesis. Considering the endothelin as a target would foster future therapeutic indications in the management of Alzheimer's disease and other neurodegenerative diseases such as Parkinson's disease. Further studies need to be explored for a clearer picture of the role of endothelin in neurodegeneration.

## Figures and Tables

**Table 1:** Various endothelin receptor agonists and antagonists on which preclinical studies are done and that are found to be effective in the prevention of Alzheimer's disease.

S. no	Drug	Agonist/antagonist	Mechanism of action	Reference
1	Bosentan	Endothelin receptor antagonist	Decrease inflammatory mediators and vascular dysfunction in brain carotid arteries, hence, decrease cerebrospinal blood flow	[48]
2	Tezosentan	ETA/ETB receptor antagonist	Reduce oxidative stress	[13]
3	BQ123	ETA/ETB receptor antagonist	Reduce oxidative stress	[13]
4	BMS 182874	ETA/ETB receptor antagonist	Reduce oxidative stress	[13]
5	IRL-1620 0.6 *μ*g/kg	Endothelin receptor agonist	Decreased oxidative stress, increased VEGF and NGF. Direct stimulation of endothelin receptor B. Antiapoptotic factor is shown against neurotoxicity by inhibiting L-type voltage-sensitive calcium channel (VSCCs)	[51, 103–105]
7	BQ123	ETA/ETB receptor antagonist	Reduce oxidative stress	[13]
8	BMS 182874	ETA/ETB receptor antagonist	Reduce oxidative stress	[13]
9	ABT-627	Endothelin A receptor (ETA) antagonist	Hinders the vasospasm activity of endothelin 1 (ET1) and nitric oxide synthesis	[54]
10	Zibotentan (ZD4054)	Endothelin A receptor antagonist or endothelin 1 receptor precursor antagonist	Improve cerebral artery blood flow or cerebral oxygenation indirectly	[5]
11	Ambrisentan	Endothelin receptor antagonists (ERAs)	Slow down pulmonary arterial hypertension progression in Alzheimer	[106]
12	Macitentan	Endothelin receptor antagonists (ERAs)	Slow down pulmonary arterial hypertension progression in Alzheimer	[106]
